# Cross-sectional study of influenza trends and costs in Malaysia between 2016 and 2018

**DOI:** 10.1371/journal.pone.0301068

**Published:** 2024-03-22

**Authors:** Sharifa Ezat Wan Puteh, Mohd Shafiq Aazmi, Muhammad Nazri Aziz, Noor ‘Adilah Kamarudin, Jamal I-Ching Sam, Ravindran Thayan, Wan Rozita Wan Mahiyuddin, Wan Noraini Wan Mohamed Noor, Adelina Cheong, Clotilde El Guerche-Séblain, Jean Khor, Eva Nabiha Zamri, Jia-Yong Lam, Zamberi Sekawi

**Affiliations:** 1 Department of Community Health, Pusat Perubatan Universiti Kebangsaan Malaysia, Kuala Lumpur, Malaysia; 2 School of Biology, Faculty of Applied Sciences, Universiti Teknologi MARA, Shah Alam, Selangor, Malaysia; 3 Lablink (M) Sdn. Bhd, Kuala Lumpur, Malaysia; 4 Department of Medical Microbiology, Faculty of Medicine, University Malaya, Kuala Lumpur, Malaysia; 5 Infectious Disease Research Centre, Institute for Medical Research, Ministry of Health Malaysia, Putrajaya, Malaysia; 6 Environmental Health Research Centre, Institute for Medical Research, Ministry of Health Malaysia, Putrajaya, Malaysia; 7 Disease Control Division, Ministry of Health Malaysia, Putrajaya, Malaysia; 8 Medical Department, Sanofi, Petaling Jaya, Malaysia; 9 Global Vaccine Epidemiology and Modelling Department, Sanofi, Singapore, Singapore; 10 Department of Medical Microbiology, Faculty of Medicine and Health Sciences, Universiti Putra Malaysia, Serdang, Selangor, Malaysia; Carol Davila University of Medicine and Pharmacy, ROMANIA

## Abstract

**Background and objectives:**

While influenza circulates year-round in Malaysia, research data on its incidence is scarce. Yet, this information is vital to the improvement of public health through evidence-based policies. In this cross-sectional study, we aimed to determine the trends and financial costs of influenza.

**Methods:**

Data for the years 2016 through 2018 were gathered retrospectively from several sources. These were existing Ministry of Health (MOH) influenza sentinel sites data, two teaching hospitals, and two private medical institutions in the Klang Valley, Malaysia. Expert consensus determined the final estimates of burden for laboratory-confirmed influenza-like illness (ILI) and severe acute respiratory infection (SARI). Economic burden was estimated separately using secondary data supplemented by MOH casemix costing.

**Results:**

Altogether, data for 11,652 cases of ILI and 5,764 cases of SARI were extracted. The influenza B subtype was found to be predominant in 2016, while influenza A was more prevalent in 2017 and 2018. The distribution timeline revealed that the highest frequency of cases occurred in March and April of all three years. The costs of influenza amounted to MYR 310.9 million over the full three-year period.

**Conclusions:**

The study provides valuable insights into the dynamic landscape of influenza in Malaysia. The findings reveal a consistent year-round presence of influenza with irregular seasonal peaks, including a notable influenza A epidemic in 2017 and consistent surges in influenza B incidence during March across three years. These findings underscore the significance of continuous monitoring influenza subtypes for informed healthcare strategies as well as advocate for the integration of influenza vaccination into Malaysia’s national immunization program, enhancing overall pandemic preparedness.

## Introduction

Epidemiological data on influenza in tropical countries is scarce. While morbidity and mortality are often high, the disease is frequently under-reported in Southeast Asian countries [1–3]. Although there have been several previous epidemiological studies on influenza in Southeast Asia, these have principally focused on pediatric populations [3, 4]. Therefore, influenza rates in adults have tended to be estimated figures. Nevertheless, the extrapolated data suggests that Southeast Asia has one of the highest influenza mortality rates in the world (3.5–9.2/100,000) [5].

In Malaysia, influenza is seen year-round, with no clear seasonal trends, and there is limited published data on its incidence [3, 6]. Published research on influenza in Malaysia is largely outdated or concerned solely with the 2009 pandemic, and samples used are not representative of the general population [3, 6–10]. The Malaysian Influenza Surveillance System was updated in 2015 and became the Malaysian Influenza Surveillance Protocol (MISP). The Ministry of Health (MOH) gathers limited data (patients’ ages and sites of origin) on laboratory-tested cases of influenza-like illness (ILI) and severe acute respiratory infection (SARI) [7, 11]. An understanding of the economic burden of disease is required to make evidence-based assessments of public health interventions [12]. Research on the 2009 influenza H1N1 pandemic has estimated total direct healthcare costs for each hospitalized patient of US$510 at a Malaysian teaching hospital [13]. This is 44% higher than Malaysia’s national expenditure on health per capita of US$353 in 2009 [3, 13, 14].

This study was conducted to determine the trends and costs of influenza in Malaysia. Using retrospective data collection, we report rates of laboratory-confirmed influenza cases and the demographic distribution of its incidence over a period of three years (2016 to 2018). We also describe the annual distribution of influenza subtypes and lineages. Particular attention was given to the economic burden of influenza, and we have therefore estimated the national fiscal costs of the disease based on the retrospective data. This is essential information in the development of effective, evidence-based health policies.

## Materials and methods

### Study design and locations

This was a cross-sectional study using retrospective data collected from five locations in Klang Valley, Malaysia. This study was conducted in accordance with the tenets of the Declaration of Helsinki 1964. The study protocol was reviewed and approved by the Ministry of Health Medical Research and Ethics Committee (Ref. No.: NMRR-19-971-46148), and the KPJ Clinical and Research Ethics Review Committee. The Ministry of Health Medical Research and Ethics Committee confirmed that the study was not more than minimal risk and only involves survey and secondary data, and hence does not require any consent as data were anonymized. Data were collected from secondary data sources for the years 2016 to 2018, inclusive using the appended form, “Survey on the health burden of influenza in Malaysia” (S1 File). These secondary data sources are cost-effective resources for epidemiological research [15]. Three types of secondary data were considered for influenza burden estimates as per the recommendations of the World Health Organization (WHO): data from SARI sentinel sites; data from ILI sentinel sites; and data from the records of hospitals not designated as SARI sentinel sites for patients admitted with acute lower respiratory infections [16]. The definition of ILI and SARI were those established by the WHO [17]. Specifically, ILI cases were identified based on the WHO case definition for ILI, which includes individuals presenting acute respiratory infection with measured fever of ≥38°C and cough, with onset within the last ten days. On the other hand, SARI cases were identified using the WHO case definition for SARI, which encompasses individuals with an acute respiratory infection, presenting with a history of fever or measured fever of ≥38°C, and cough, with onset within the last ten days, and a requirement for hospitalization due to the severity of their condition. The specific sites from which data were collected were the following: the Virology Unit of the Institute of Medical Research (IMR), Setia Alam; the National Public Health Laboratory (NPHL), Sungai Buloh; the University of Malaya Medical Center (UMMC), Kuala Lumpur; Kumpulan Perubatan Johor (KPJ) Hospitals (Damansara and Ampang Puteri), Kuala Lumpur; and Hospital Canselor Tuanku Muhriz Universiti Kebangsaan Malaysia (HCTM UKM), Kuala Lumpur.

IMR’s Virology Unit is a WHO National Influenza Center (NIC) in Malaysia with influenza data from the whole country. Among the influenza surveillance carried out at this site is the weekly collection of five samples each from eight sentinel sites spread across Malaysia. The samples are taken from patients tested at each site for SARI and were subjected to the gold-standard real-time reverse transcription-polymerase chain reaction (RT-PCR) method in IMR for the detection and subtyping of the virus.

The NPHL monitors influenza incidence through ILI surveillance. The center receives five samples weekly from 15 ILI sentinel sites across Malaysia. Typically, for each patient, NPHL receives two samples from the sentinel sites, which include nasopharyngeal and oropharyngeal swabs in viral transport media (VTM). These were then combined during the processing stage and were treated as a single sample from the respective patient during the analysis. Similar to the practice in IMR, RT-PCR was conducted in NPHL on the samples to detect and subtype the influenza virus.

The UMMC is another designated WHO NIC. Data from this site were from diagnostic test samples collected from patients admitted to UMMC. In contrast to the other sites, influenza was detected using immunofluorescence assay and viral culture in UMMC. The private KPJ Hospitals (Damansara and Ampang Puteri) were included to present the burden and costs of influenza in private rather than public healthcare facilities in Malaysia. Similar to IMR and NPHL, the gold-standard RT-PCR method was also performed in KPJ Hospitals. HCTM UKM was included to assist with the estimation of the financial costs of influenza. As all three hospitals recorded the primary International Classification of Diseases (10th edition) (ICD-10) diagnostic codes for each patient on discharge, we were able to gather all influenza patient data from these hospitals by identifying those patients with the relevant ICD-10 codes (S2 File). For sensitive surveillance purposes, influenza cases at these hospitals were defined as either inpatients or outpatients with an ICD-10 diagnosis of “influenza-like-illness” (the specific ICD codes are appended in the S2 File) [16].

### Data extraction

In the presence of heterogeneous findings from the primary data collection, additional extrapolation analyses and expert consultation supported in filling missing research gaps to enhance the estimation of health and economic burdens associated with laboratory-confirmed cases of SARI and ILI over three years in Malaysia.

The incidence rates for influenza cases that received medical attention during the period studied were estimated based on combination of the data sources described above. To ensure all cases were captured, two denominator populations were used to assess the influenza population in Malaysia: those diagnosed with ILI and those diagnosed with SARI. Expert committee members agreed on the definitions and diagnoses, extracted from the relevant ICD-10 codes, of these two conditions, to identify relevant cases in the “casemix” system patient database of the Malaysia MOH. These cases were then collated, tabulated, and further clarified based on laboratory confirmations of influenza.

Casemix is a system used in Malaysia that combines hospitals’ clinical and demographic patient data and allows variable-based classification and categorization of specified patient groups for data collation purposes. It was first used in Malaysia in HCTM UKM and has been since then implemented in MOH hospitals throughout the country. It is known as MyDRG and is largely used as a costing tool [18]. The MyDRG codes for influenza patients during the period studied were extracted along with the patient management costs from the wards. Information regarding comorbidities, length of hospitalization, and severity of illness was also collected. Using these diagnosis-related group (DRG) codes, it was possible to establish the estimated financial costs of hospitalized influenza cases. However, this approach is limited to direct medical costs from healthcare providers. Indirect economic losses resulting from morbidity (the value of lost productivity by persons either unable to perform their usual work tasks or able to do so less effectively due to the illness) and mortality (the value of lost productivity due to deaths from the illness) were not taken into account as both data collection and accurate calculations for these are difficult to attain.

### Data analysis

Descriptive statistics showing the frequency of influenza-positive laboratory results among both the ILI and SARI patient samples and categorized according to variables that included study sites, age groups, gender, and year were determined. A lack of information prevented the analysis of clinical variables such as comorbidities, vaccination history, and mortality. A similar approach was applied with the data stratified by influenza subtypes (influenza A, influenza B, or dual influenza A and B infection). To investigate influenza trends and seasonality of influenza in Malaysia, time series analysis was performed using the percentage of influenza-positive cases sampled across each of the three years studied. These were presented as a time series plot. All analyses were performed using SPSS^®^ v. 22.0 (IBM, USA) software and Microsoft Excel 2016 (Microsoft, USA).

The MyDRG data system was not implemented in two study sites: UMMC and KPJ. However, both centers used ICD-10 coding of medical conditions. Hence, to determine the costs for these two sites, the relevant ICD-10 codes were used instead of MyDRG. The ICD-10 codes, length of hospital stay (in days), and other diagnoses were entered into the MyDRG software to produce the DRG codes. The data were used to estimate the inpatient cost of SARI at four sites: HCTM UKM, MOH, UMMC, and KPJ. Outpatient (ILI) treatment costs were estimated from data for two study sites (NPHL and KPJ) using the per-patient costing data methodology from previous research [19]. The overall yearly cost was then determined based on the number of cases per site each year.

## Results

### Distribution of influenza cases across baseline characteristics

The descriptive statistics of influenza-positive cases, divided by ILI and SARI are presented in Table 1. The total number of clinical cases included in the study, combining both ILI and SARI cases, was 17,416. The majority of these were ILI (66.9%). Most of the ILI samples were from the NPHL (89.7%), whereas most of the SARI samples were from IMR (53.7%), followed by UMMC (25.4%) and KPJ (20.8%). The influenza positivity rate of amongst SARI samples was highest in those from KPJ (18.3%) followed by IMR (16.1%) and UMMC (5.6%). Similarly, the positivity rate among the ILI samples was also highest for KPJ (18.2%) samples followed by NPHL (15.3%).

**Table 1:** Distribution of influenza according to baseline characteristics (n = 17,416).

Characteristics	ILI		SARI	
	Influenza-positive		Influenza-positive	
	n	%	n	%
**Site**				
NPHL	1,601/10,452	15.3%	NA	NA
KPJ	218/1,200	18.2%	219/1,200	18.3%
IMR	NA	NA	500/3,098	16.1%
UMMC	NA	NA	82/1,466	5.6%
**Age** ^a^				
<2 years	83/1,056	7.9%	187/2,462	7.6%
2–4 years	161/1,215	13.3%	128/826	15.5%
5–14 years	704/3,255	21.6%	81/426	19.0%
15–49 years	769/5,106	15.1%	128/689	18.6%
50–64 years	79/733	10.8%	104/496	21.0%
>64 years	23/283	8.1%	117/617	19.0%
**Gender** ^b^				
Male	988/5,998	16.5%	404/3,269	12.4%
Female	827/5,603	14.8%	396/2,493	15.9%
**Year**				
2016	513/3,560	14.4%	180/1,593	11.3%
2017	786/4,311	18.2%	318/2,065	15.4%
2018	520/3,781	13.8%	303/2,106	14.4%

IMR, Institute of Medical Research; KPJ, Kumpulan Perubatan Johor Hospitals; NA, not applicable; NPHL, National Public Health Laboratory; UMMC, University of Malaya Medical Center.

^a^ 252 samples were excluded owing to missing age information.

^b^ 53 samples were excluded owing to missing gender information.

The number of ILI samples outweighed the number of SARI samples across all age groups, except for those under <2 years old and those over >64 years old. The distribution of ILI across age groups indicated the highest rates among 5- to 14-year-olds (with a positivity rate of 21.6%) and decreasing rates with lower and higher age. A comparable trend was seen for SARI, with low rates of positive samples in those under 5 but consistently high rates in those older than 5, peaking at 21.0% in 50- to 64-year-olds.

In terms of gender, the proportion of male samples (53.2%) was more than female samples (46.5%). ILI samples contributed to most samples across gender. In terms of the percentages of confirmed influenza cases, both the positivity rate is almost comparable between male (15.0%) and female (15.1%) samples. When the data is stratified based on ILI and SARI status, there are more than 50% of confirmed influenza cases in males and females from ILI.

The highest number of clinical samples received occurred in 2017 (36.6%) followed by 2018 (33.8%), and 2016 (29.6%). Over the entire three years, there were more ILI samples (66.9%) than SARI samples (33.1%). A similar trend was observed in positivity rates, with the number of laboratory-confirmed influenza cases highest in 2017 (17.3%) followed by 2018 (13.9%) and (13.5%).

### Trends in the incidence of influenza

**Fig 1:**
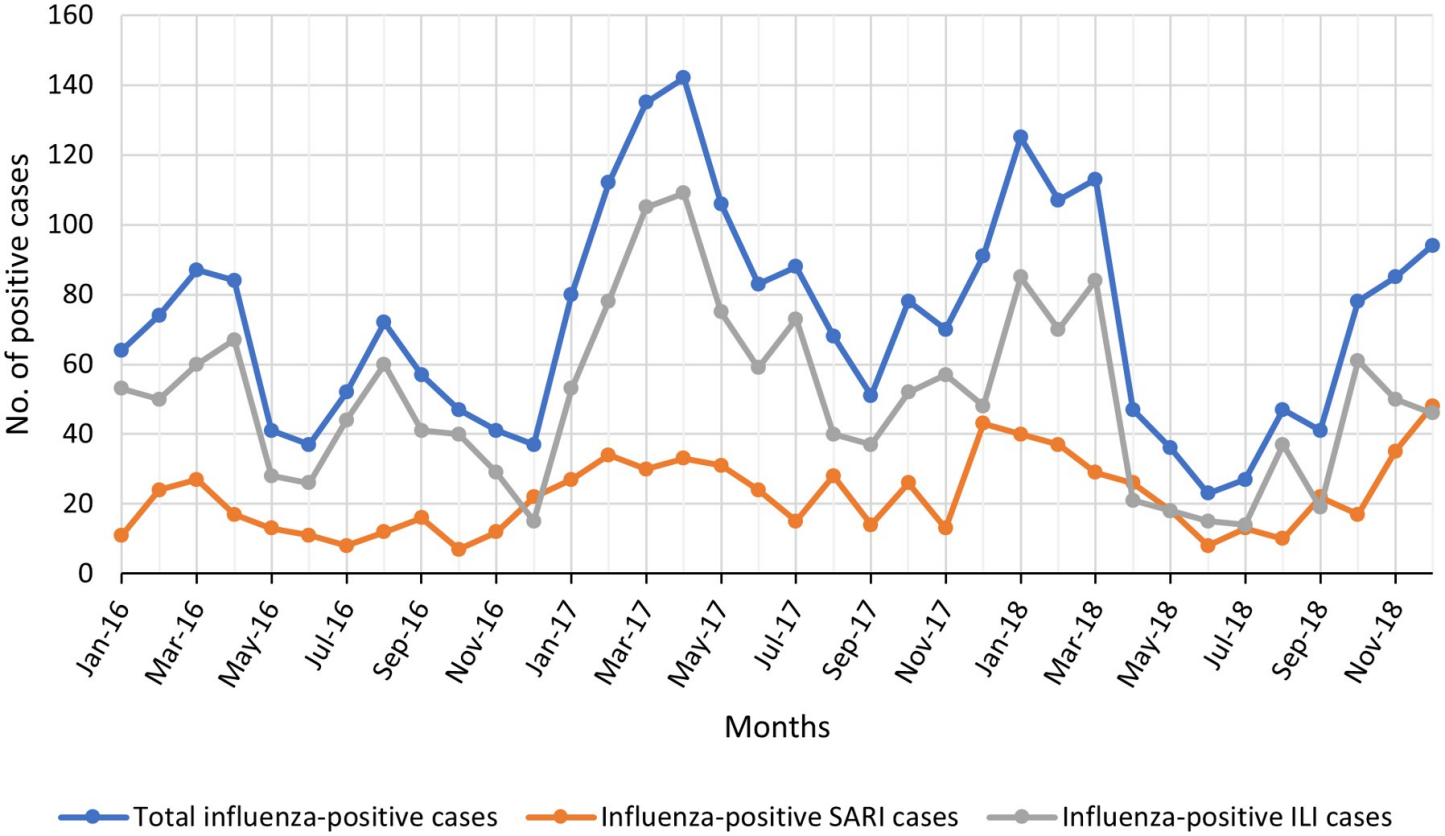
Graph showing overall trends in influenza cases from 2016 to 2018.

Trend analysis of positive influenza cases throughout the three years found clear seasonality pattern, with systematic peaks and troughs in certain months (Fig 1). The combined incidence rate of ILI and SARI cases exhibits notable peaks in March-April across the three-year period (2016–2018). Additionally, there is an extra peak observed in August 2016, which does not recur in the following years. A greater number and percentage of positive cases were noted in 2017 than in 2016 or 2018.

When the trend was separated into positive cases among SARI samples (Fig 1), there was a general decrease each year between February and August in all three years, and an increase from August to December. However, small peaks were noted in August and October of 2017 that contrasted with the data from 2016 and 2018. Additionally, it is crucial to highlight that the early-year peak (January to February) actually represents a continuation of a rising trend that commenced from the end of the preceding year. In the ILI samples (Fig 1), there was a trend of decrease from January to December in all years and systematic peaks in March to April of 2016 and 2017, but not in 2018. Small peaks were also observed in July 2017, August 2016, and August 2018. These findings align with the trends in the percentage of positive cases for SARI and ILI observed over the three-year period when displayed as stacked months (S1 Fig).

### Distribution of influenza subtypes

**Fig 2:**
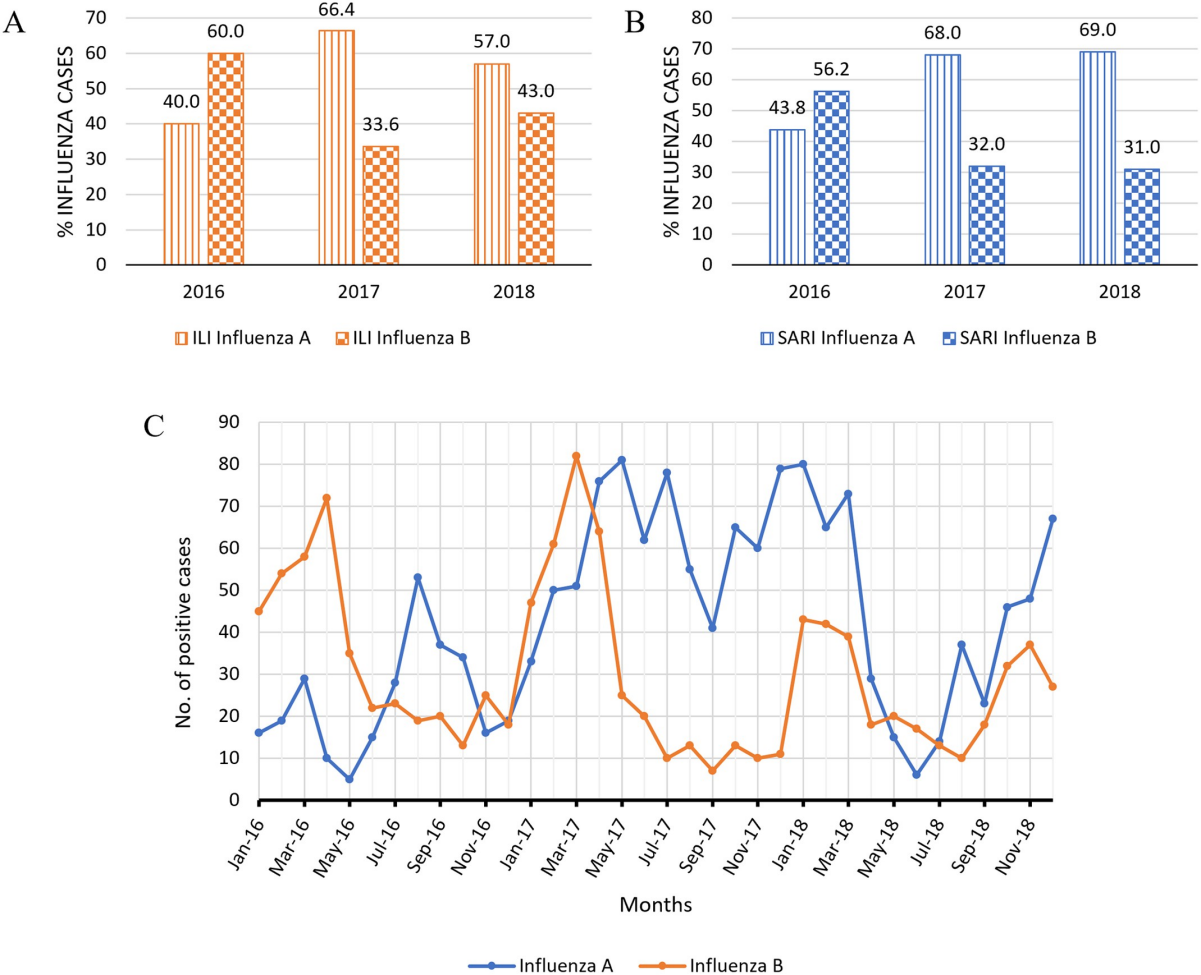
Distribution of influenza subtypes. (A) Distribution of influenza subtypes by year in laboratory-confirmed cases of influenza-like illness. (B) Distribution of influenza subtypes by year in laboratory-confirmed cases of severe acute respiratory infection. (C) Chronological graph showing overall trends in incidence rates of the A and B influenza subtypes in 2016, 2017, and 2018.

When the ILI and SARI cases were categorized by influenza virus subtype, influenza B was found to be the predominant type in 2016 among the ILI samples (60.0%), while influenza A constituted the highest percentages in 2017 and 2018 (66.4% and 57.0%, respectively) (Fig 2A). Similarly, most cases identified in the SARI samples in 2016 were influenza B (56.2%). As in the ILI samples, influenza A was the predominant subtype in 2017 and 2018 among the SARI samples (68.0% and 69.0%, respectively) (Fig 2B). Over the three years, trends in the percentages of positive cases of each virus subtype showed more peaks of higher magnitude for cases of influenza A (Fig 2C). The series also identified an influenza A epidemic in 2017, shown in frequent upsurges in the number of positive cases of this virus subtype. Influenza B incidence consistently peaked in March throughout the three years.

### Influenza cost estimation

Economic burden was estimated separately for ILI and SARI as the two have relatively heterogeneous costs. All ILI patients included in our study were outpatients; and per WHO definition, all SARI patients were hospitalized. Using the data from our three-year study period from three of the treatment sites (two KPJ hospitals and NPHL), we calculated the average costs per case for ILI treatment in both the public and private sectors. These costs are summarized in Table 2. The price per case (PPC) for ILI treatment was based on a previous study [19]. The total costs of treating upper respiratory tract infection outpatients in the public and private sectors were MYR 43.23 and MYR 60.45, respectively [19]. Total ILI spending by both sectors amounted to MYR 524,380, with NPHL (public) having higher overall ILI treatment costs (MYR 451,840) than KPJ (private) owing to the greater number of cases treated in the public sector.

**Table 2:** Total costs of ILI treatment in the public (NPHL) and private (KPJ) sectors from 2016 to 2018.

ILI sites	PPC (MYR)	Number of cases	Total price (MYR)
NPHL	43.23	10,452	451,840
KPJ	60.45	1,200	72,540
**Total**		**11,652**	**524,380**

ILI, influenza-like illness; KPJ, Kumpulan Perubatan Johor Hospitals; MYR, Malaysian ringgit; NPHL, National Public Health Laboratory; PPC, price per case.

Table 3 shows the background information of the health costs of SARI attained from the MOH casemix system. As described earlier, the ICD codes for some of the samples from UMMC and KPJ gave upper respiratory tract infection or influenza as a secondary diagnosis. As such, the conversion of these sample codes to DRG did not place them in the DRG categories for respiratory infections but grouped them as ear, nose, and throat (ENT) or orbital infections, and blood disorders. However, those samples categorized in this way that tested positive for influenza still warranted inclusion in our dataset. To avoid resulting inaccuracies in our influenza costing, we maintained the grouping of these cases by their primary diagnoses, as shown in S2 Table. The PPC for respiratory infections, ENT and orbital infections, and blood disorders was dependent on the illness severity level, average length of hospital stay (ALOS), and case group weight (CGW). Generally, higher severity levels result in lengthier hospital stays, higher CGW, and higher PPC. The PPC costing methodology incorporated the costs of repeated hospital visits and readmissions over the entire three years.

**Table 3:** Severe acute respiratory infection treatment costs per case (from 2016 to 2018) based on severity levels, average length of hospital stays, case group weights, and national base rates.

DRG codes^a^	ALOS (days)	CGW	National base rate^b^ (MYR)	PPC (MYR)
**Respiratory infection**				
4581	5.11	1.006	4,063.10	4,088.30
4582	4.67	0.954	4,063.10	3,876.39
4583	6.75	1.329	4,063.10	5,400.95

ALOS, average length of stay; CGW, case group weight; DRG, diagnosis-related group; MYR, Malaysian ringgit; NBR, national base rate; PPC, price per case; SARI, severe acute respiratory infection.

^a^ DRG code 4581: respiratory failures with lowest severity of illness; DRG code 4582: respiratory failures with moderate severity of illness; DRG code 4583: respiratory failures with highest severty of illness.

^b^ National base rate is a predetermined amount payable for treatment of a patient based on DRG averages and before any adjustments to weight.

The PPC for SARI treatment was determined (Table 3) and SARI treatment costs for the different respiratory infection DRG codes were calculated for the MOH sites (Table 4). The cost of severity level 1 was the highest at MYR 252.6 million as the majority of cases were classed at this severity level. Collectively, the total cost of SARI treatment in MOH sites amounted to MYR 287.4 million. The SARI treatment costs were also calculated for HCTM UKM, UMMC, and KPJ (S3 Table). Data from UMMC and KPJ were combined as these two sites did not have the DRG software but used ICD-10 diagnostic codes. The total cost of SARI treatment across all sites and DRG codes amounted to MYR 310.3 million. Collectively, the combined cost of ILI (MYR 524,380) and SARI (MYR 310.3 million) for all cases and severity levels across all study sites and all three years studied was MYR 310.9 million.

**Table 4:** Costs of severe acute respiratory infection treatment in MOH sites.

DRG codes	PPC (MYR)	Number of cases	Total price (MYR)
**Respiratory infection**			
4581	4,088.30	61,774	252.6 million
4582	3,876.39	4,164	16.1 million
4583	5,400.95	3,470	18.7 million
**Total**		69,408	287.4 million

DRG, diagnosis-related group; MYR, Malaysian ringgit; MOH, Ministry of Health; PPC, price per case; SARI, severe acute respiratory infection.

## Discussion

Overall, the present study analyzed results from 11,652 ILI and 5,764 SARI samples, showing influenza rates of 15.6% and 13.9%, respectively. This yielded 2,620 (15.0%) laboratory-confirmed cases of influenza from 17,416 samples, higher than the previously reported rate (4.8% to 10.3%) in Malaysia [3, 20, 21]. The difference between the percentage of samples from UMMC that tested positive for SARI and the percentages found in the samples from other sites could be due to lack of systematic disease screening such as reverse transcription-polymerase chain reaction (RT-PCR) tests. At the IMR, NPHL, and KPJ sites, the use of confirmatory PCR diagnostic tests is standard practice. However, patient samples taken at the UMMC are generally subjected to a less sensitive immunofluorescence assay, followed by virus isolation. It is worth noting that the exclusion of data from the UMMC site did not substantially alter the overall dataset findings (S1 Table).

Among all sites, we observed (unrelated to the influenza-positive rates among samples) a lower frequency of samples from older adults. Possible reasons for this may include site inaccessibility in older people with reduced mobility or transport issues; lack of motivation to seek treatment, concerns about treatment, healthcare settings, miscommunication, and lack of emotional support [22–24]. The discrepancy may also have socioeconomic causes; older adults whose adult children have the financial means may prefer to have their parents treated at specialist private clinics. Introduction of free public healthcare for senior citizens in 2013 had left many public hospitals overcrowded and overstretched, potentially reducing the quality of care [25].

Over the study period, influenza B rates were higher in 2016 and influenza A in 2017 and 2018. However, the timeline of the influenza subtype distributions showed two changes to the predominant type in circulation in 2016: from influenza B to influenza A and then back to B. This aligns with a contemporaneous study also using Malaysia Influenza Surveillance System and UMMC data [20]. The distribution timeline of their study period revealed that seasonal transmission occurs either from September to April or from June to August. This is congruent with previous reports indicating that the presence or absence of seasonal peaks in Malaysia is unpredictable [2, 20, 26]. Influenza infections are reported throughout the year in tropical and sub-tropical Southern and Southeast Asian countries, but there are generally two significant outbreaks annually. Despite consistent climate conditions, outbreak timing varies across these countries [27]. The proximity and extent of contact between people in a location affect the scale of any influenza epidemic, with increased incidence rates in areas where people congregate [28]. In Malaysia and Singapore, holidays tend to take place toward the end of the year, at which time, many people travel abroad, potentially bringing new strains on their return. Longitudinal seroepidemiological studies in Singapore and Hong Kong have identified negative correlations between waves of seasonal influenza activity and population immune resistance [29]. The interplay of antigenic drift and waning immunity post-infection and/or vaccination likely drives outbreak timing in the tropics.

While the present study sheds light on influenza patterns during 2016 to 2018, it is important to acknowledge the dynamic of the influenza landscape beyond this period. Towards late 2018, a notable increase in positive cases was noted, primarily attributed to influenza A. This aligns with the findings of Pang et al. (2021), reporting on a prospective epidemiological active surveillance study in the Klang Valley area of Malaysia from July 2018 to August 2019, in which a surge in the influenza-positive rate was identified in November 2018, coinciding with A/H3N2 becoming the predominant strain for approximately two months. Subsequently, A/H1N1pdm09 emerged as the primary strain from January 2019, maintaining its predominance until the conclusion of the study in August 2019. The heightened influenza-positive rates were notable between November 2018 and February 2019 and again from June to July 2019 [21]. These findings correspond with data documented by FluNet, a web-based influenza surveillance system hosted by the World Health Organization. Analysis of FluNet data shows another significant increment in late 2019, peaking between December 2019 and January 2020, followed by a sharp decline in March 2020, coinciding with the surge of the COVID-19 pandemic. Tan et al. (2022) conducted a multi-center, prospective, observational study among individuals aged 65 years and over who presented with SARI at four Malaysian hospitals from January 2021 to December 2021, and interestingly, they detected only one case of influenza, alongside a high SARS-CoV-2 positively rate. As suggested, infection control measures such as lockdowns and physical distancing implemented to mitigate the spread of SARS-CoV-2 also had a profound impact on influenza transmission in numerous countries during 2020 and 2021 with notably low incidence of influenza infections [30]. Interestingly, FluNet data for late 2021 revealed resurgence of influenza, characterized by a spike reaching 12% of influenza-positive specimens, primarily attributed to influenza A in January 2022. This resurgence was followed by another substantial spike, peaking at 37% in July 2022. In 2022, yet another surge was observed, primarily associated with influenza B, spanning from October 2022 to March 2023, with a peak in January 2023 at 36%. Collectively, these highlight the dynamic nature of influenza trends, emphasizing the need for continuous surveillance and research to facilitate a more informed public health decision-making.

Influenza vaccination uptake is generally low in Malaysia [21]. As influenza activity in tropical countries is usually complex and unpredictable [20], long-term analysis of national and regional data is crucial for effective public health interventions. However, recent comparison of the influenza surveillance systems of Australia, China, and Malaysia identified several gaps in the Malaysian surveillance system [31]. Improvements to the national influenza surveillance system should include expansion of sentinel networks and increasing the use of RT-PCR tests to better characterize the national burden and map the circulation of subtype infections. Although the current surveillance data were categorized into age groups, these were not based on WHO guidance. The lack of available information on underlying conditions and immunization status was also a hindrance to comprehensive burden estimates, and this data should also be made available. This would help generate more reliable data for more well-informed policy decisions and greater influenza protection of high-risk groups.

Future research on the burden of influenza in Malaysia could support the findings of this study. There were several limitations to this study. The retrospective nature of the data raises concerns about the quality and adequacy of medical records, potentially leading to underreporting. The calculation of population-based rates is complicated, which is inherent to the present approach. There were variations in the number of samples contributed by each site included in this study. Surveillance samples were sent weekly from each site but, since this was at their discretion, there was a degree of variation in sample numbers. From a health economics perspective, only the direct core costs from healthcare providers could be ascertained, as primary data for indirect costs were difficult to obtain. Hospitalization costs were estimated based on the MOH casemix system implemented in MOH hospitals throughout Malaysia, but two hospitals (UMMC and KPJ) lacked this system, limiting data availability on hospitalization costs per case. Notwithstanding the limitations, it is vital to obtain a preliminary understanding of the epidemiology of influenza in Malaysia.

## Conclusions

In summary, our findings demonstrated the presence of influenza year-round in Malaysia and, while seasonal peaks do occur, they are inconsistent. We also found evidence for an influenza A epidemic in Malaysia in 2017 and noted a consistent surge in influenza B incidence rates in March throughout the three years. Mapping the distribution and incidence of influenza A and B is pivotal to effective healthcare decisions and the timing of vaccine administration. The current data suggest that incorporating seasonal influenza vaccination into Malaysia’s national immunization program is a crucial step, not only in mitigating seasonal influenza but also in strengthening preparedness for potential pandemics, reducing the risk of co-infections. These data will enhance understanding of influenza in Malaysia and help define public health research and policy priorities.

## Supporting information

S1 File Survey on the health burden of influenza in Malaysia. (PDF)

S2 File ICD-10 codes applied for surveillance. (PDF)

S1 Fig Monthly trends of influenza incidence. (A) Monthly trends over three years in the incidence of severe acute respiratory among the samples in this study. (B) Monthly trends over three years in the incidence of influenza-like illness among the samples in this study.
(PDF)

S1 Table Distribution of influenza according to baseline characteristics (n = 15,950, UMMC excluded). (PDF)

S2 Table Severe acute respiratory infection treatment costs per case based on DRG codes, severity levels, average length of hospital stays, case group weights, and national base rates. (PDF)

S3 Table Costs of severe acute respiratory infection treatment at four Malaysian treatment sites. (PDF)
