# Supplementary material for: Cross-sectional study of influenza trends and costs in Malaysia between 2016 and 2018
Source: PLoS One. 2024 Mar 22;19(3):e0301068. doi: 10.1371/journal.pone.0301068 (PMC10959333; doi:10.1371/journal.pone.0301068)
Supplement: S1 File — (PDF) [file pone.0301068.s001.pdf]

S1 File. Survey on the health burden of influenza in Malaysia.

SURVEY ON THE HEALTH BURDEN OF INFLUENZA IN MALAYSIA (KAJIAN BEBAN KESIHATAN INFLUENZA DI MALAYSIA)

GENERAL INFORMATION

1. Patient MRN #:____
2. Hospital: ____
3. Date of birth: [ ] / [ ] / [ ]
4. Race: ____
5. IC Number: [ ] - [ ] - [ ]
6. Gender:

   [ ] Male
   [ ] Female

7. Patient Address:

   ____

   ____

8. Patient contact information (Tel. no): _____________________________
9. DOA (Date of Admission): [ ] / [ ] / [ ]
10. DOD (Date of Discharge): [ ] / [ ] / [ ]
11. LOS (Length of Stay): _____ days
12. Ward: ____
13. Outcome:

    [ ] Discharge
    [ ] Death

14. Fever ≥38°C or history of fever for a few days prior to hospital visit :

    [ ] Yes [ ] No [ ] Unknown

15. Cough :

    [ ] Yes [ ] No [ ] Unknown

16. X Ray Findings: ____

Optional Details

17. Does patient have any comorbidity? (You can tick (✓) more than one)

- [ ] None
- [ ] High Blood Pressure
- [ ] Diabetes
- [ ] High Cholesterol
- [ ] Kidney Failure
- [ ] Asthma
- [ ] Cardiovascular Disease
- [ ] Stroke
- [ ] Chronic obstructive pulmonary disease (COPD)
- [ ] Pregnancy
- [ ] Other (Please state if there is any): ________________

18. Did the patient receive seasonal influenza vaccination within the last 12 months?

- [ ] Yes
- [ ] No
- [ ] Not Known

19. Is the patient a smoker?

- [ ] Yes
- [ ] No
- [ ] Not Known

A. LABORATORY DETAILS

INFLUENZA LABORATORY INFORMATION

1. Has any Influenza Laboratory test been conducted?

☐ Yes ☐ No

2. Which type of test was done?

☐ Real Time RT-PCR or Any Form of PCR
☐ Digital Immunoassay
☐ Immunochromatographic rapid
☐ Immunofluorescence test alone + culture
☐ Immunofluorescence test
☐ Blood Culture
☐ Other (Please specify):

3. What is the confirmation date of the laboratory results? ___ / ___ / ___

4. Laboratory results:

4.1 Type of test: _______________

☐ Positive ☐ Negative ☐ Unknown

4.2 Type of test: _______________

☐ Positive ☐ Negative ☐ Unknown

4.3 Type of test: _______________

☐ Positive ☐ Negative ☐ Unknown

4.4 If the tested sample is positive, which influenza strain?

☐ Influenza A ☐ Influenza B ☐ Unknown

4.4.1 If Influenza A strain,

☐ H1N1 ☐ H3N2 ☐ Unknown

4.4.1.1 What is the CT value? ____________

4.4.2 If Influenza B strain

☐ Victoria ☐ Yamagata ☐ Unknown

4.4.2.1 What is the CT value? ____________

B. COSTING DETAILS

Notes: The following International Classification Disease (ICD): ICD9 and ICD10 codes and diagnosis-related groups (DRG) code are included in the case definition (refer to Appendix 1).

1. ICD code of interest to ILI/ARI as in the list: _____________

   ILI= Influenza-like Illness, ARI= Acute Respiratory Infection (code refer Appendix 1)

2. Primary Clinical Diagnosis (ICD code) of patient : __________

3. Secondary Clinical Diagnosis (ICD Code) of patient (*if any, can fill up to 10 code*):

   a) _______________ f) _______________

   b) _______________ g) _______________

   c) _______________ h) _______________

   d) _______________ i) _______________

   e) _______________ j) _______________

4. Primary Procedure (ICD code) of patient (*if any*) : ______________

5. Secondary Procedure (ICD Code) of patient (*if any, can fill up to 10 code*):

   a) _______________ f) _______________

   b) _______________ g) _______________

   c) _______________ h) _______________

   d) _______________ i) _______________

   e) _______________ j) _______________

6. Final Diagnostic Related Group (DRG) code for this episode of care: ________

7. Patients’ Severity Level:

☐ Severity of Illness I (SOI I)

☐ Severity of Illness II (SOI II)

☐ Severity of Illness III (SOI III)
