# Supplementary material for: Cross-sectional study of influenza trends and costs in Malaysia between 2016 and 2018
Source: PLoS One. 2024 Mar 22;19(3):e0301068. doi: 10.1371/journal.pone.0301068 (PMC10959333; doi:10.1371/journal.pone.0301068)
Supplement: S2 File — (PDF) [file pone.0301068.s002.pdf]

S2 File. ICD-10 codes applied for surveillance.

ICD-10 codes	
B97.89 (other viral agents as the cause of diseases classified elsewhere)	
H66.9 (otitis media, unspecified)	•H66.90 (otitis media, unspecified, unspecified ear)<br>•H66.91 (otitis media, unspecified, right ear)<br>•H66.92 (otitis media, unspecified, left ear)<br>•H66.93 (otitis media, unspecified, bilateral ear)
J0T.9 (acute sinusitis, unspecified)	•J01.90 (acute sinusitis, unspecified)<br>•J06.9 (acute upper respiratory infection, unspecified)
J09 (influenza due to certain identified influenza viruses)	
J09.X (influenza due to identified novel influenza A viruses)	•J09.X1 (influenza due to identified novel influenza A virus with pneumonia)<br>•J09.X2 (influenza due to identified novel influenza A virus with other respiratory manifestations)<br>•J09.X3 (influenza due to identified novel influenza A virus with gastrointestinal manifestations)<br>•J09.X9 (influenza due to identified novel influenza A virus with other manifestations)
J10 (influenza due to other identified influenza viruses)	
J10.0 (influenza due to identified novel influenza A viruses)	•J10.01 (influenza due to other identified influenza virus with the same other identified influenza virus pneumonia)<br>•J10.08 (influenza due to other identified influenza virus with other specified pneumonia)<br>•J10.81 (influenza due to other identified influenza virus with other manifestations with encephalopathy)<br>•J10.82 (influenza due to other identified influenza virus with other manifestations with myocarditis)<br>•J10.83 (influenza due to other identified influenza virus with other manifestations with otitis media)<br>•J10.89 (influenza due to other identified influenza virus with other manifestations)
J11 (influenza due to unidentified influenza virus)	
J11.0 (influenza due to unidentified influenza virus with pneumonia)	•J11.00 (influenza due to unidentified influenza virus with unspecified type of pneumonia)<br>•J11.08 (influenza due to unidentified influenza virus with specified pneumonia)
J11.1 (influenza due to unidentified influenza virus with other respiratory manifestations)	
J11.2 (influenza due to unidentified influenza virus with gastrointestinal manifestations)	
J11.8 (influenza due to unidentified influenza virus with other manifestations)	•J11.81 (influenza due to unidentified influenza virus with encephalopathy)<br>•J11.82 (influenza due to unidentified influenza virus with myocarditis)<br>•J11.83 (influenza due to unidentified influenza virus with otitis media)<br>•J11.89 (influenza due to unidentified influenza virus with other manifestations)
J12.89 (other viral pneumonia)	
J12.9 (viral pneumonia, unspecified)	
J18 (pneumonia, unspecified organism)	•J18.1 (lobar pneumonia, unspecified organism)<br>•J18.8 (other pneumonia, unspecified organism)<br>•J18.9 (pneumonia, unspecified organism)
J20.9 (acute bronchitis, unspecified)	
J40 (bronchitis, not specified as acute or chronic)	
R05 (cough)	
R50.9 (fever, unspecified)	
