# Supplementary figures and images for: Cross-sectional study of influenza trends and costs in Malaysia between 2016 and 2018
Source: PLoS One. 2024 Mar 22;19(3):e0301068. doi: 10.1371/journal.pone.0301068 (PMC10959333; doi:10.1371/journal.pone.0301068)

A

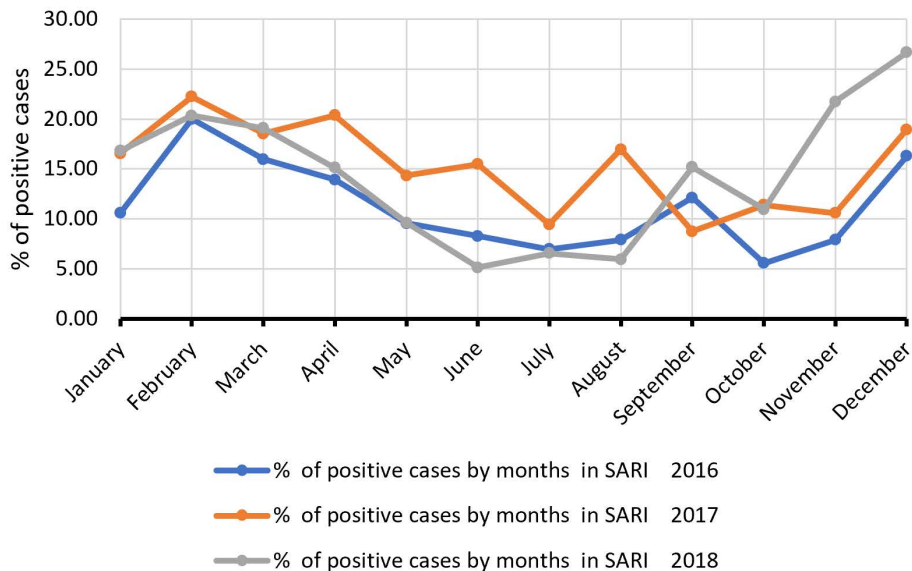

B

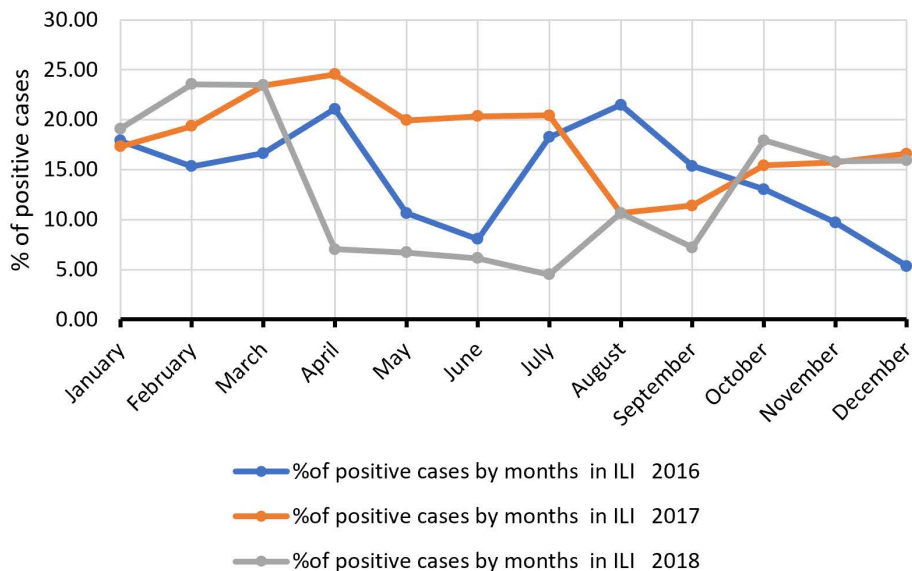

Supplement: S1 Fig — (A) Monthly trends over three years in the incidence of severe acute respiratory among the samples in this study. (B) Monthly trends over three years in the incidence of influenza-like illness among the samples in this study. (PDF) [file pone.0301068.s003.pdf]
