# Supplementary material for: Cross-sectional study of influenza trends and costs in Malaysia between 2016 and 2018
Source: PLoS One. 2024 Mar 22;19(3):e0301068. doi: 10.1371/journal.pone.0301068 (PMC10959333; doi:10.1371/journal.pone.0301068)
Supplement: S1 Table — (PDF) [file pone.0301068.s004.pdf]

S1 Table. Distribution of Influenza According to Baseline Characteristics (n = 15,950, UMMC excluded).

Characteristics	ILI		SARI		Total	
	Influenza-positive		Influenza-positive		Influenza-positive	
	n	%	n	%	n	%
**Site**						
NPHL	1,601/10,452	15.3%	NA	NA	NA	NA
KPJ	218/1,200	18.2%	219/1,200	18.3%	NA	NA
IMR	NA	NA	500/3,098	16.1%	NA	NA
**Age**[a]						
<2 years	83/1,056	7.9%	151/1,551	9.7%	234/2,607	9.0%
2–4 years	161/1,215	13.3%	94/493	19.1%	255/1,708	14.9%
5–14 years	704/3,255	21.6%	75/289	26.0%	779/3,544	22.0%
15–49 years	769/5,106	15.1%	125/653	19.1%	894/5,759	15.5%
50–64 years	79/733	10.8%	103/477	21.6%	182/1,210	15.0%
>64 years	23/283	8.1%	115/587	19.6%	138/870	15.9%
**Gender**						
Male	988/5,998	16.5%	362/2,440	14.8%	1,350/8,438	16.0%
Female	827/5,603	14.8%	356/1,856	19.2%	1,183/7,459	15.9%
Unknown	4/51	7.8%	1/2	50.0%	5/53	9.4%
**Year**						
2016	513/3,560	14.4%	164/1,140	14.4%	677/4,700	14.4%
2017	786/4,311	18.2%	284/1,559	18.2%	1,070/5,870	18.2%
2018	520/3,781	13.8%	271/1,599	17.0%	791/5,380	14.7%

IMR, Institute of Medical Research; KPJ, Kumpulan Perubatan Johor Hospitals; NA, not applicable; NPHL, National Public Health Laboratory.

[a] 252 samples were excluded owing to missing age information.
