# Supplementary material for: Cross-sectional study of influenza trends and costs in Malaysia between 2016 and 2018
Source: PLoS One. 2024 Mar 22;19(3):e0301068. doi: 10.1371/journal.pone.0301068 (PMC10959333; doi:10.1371/journal.pone.0301068)
Supplement: S2 Table — (PDF) [file pone.0301068.s005.pdf]

S2 Table. Severe Acute Respiratory Infection Treatment Costs per Case Based on DRG Codes, Severity Levels, Average Length of Hospital Stays, Case Group Weights, and National Base Rates.

DRG codes	ALOS (days)	CGW	National base rate[a] (MYR)	PPC (MYR)
**Respiratory infection**				
4581	5.11	1.006	4,063.10	4,088.30
4582	4.67	0.954	4,063.10	3,876.39
4583	6.75	1.329	4,063.10	5,400.95
**ENT and orbital infection**				
3501	2.98	0.579	4,063.10	2,356.40
3502	3.39	0.627	4,063.10	2,549.70
3503	4.27	0.818	4,063.10	3,325.10
**Blood disorder**				
16501	3.23	0.616	4,063.10	2,505.86
16502	4.01	0.79	4,063.10	3,211.70
16503	5.57	1.148	4,063.10	4,664.50

ALOS, average length of stay; CGW, case group weight; DRG, diagnosis-related group; ENT, ear, nose, and throat; MYR, Malaysian ringgit; NBR, national base rate; PPC, price per case; SARI, severe acute respiratory infection.

[a] National base rate is a predetermined amount payable for treatment of a patient based on DRG averages and before any adjustments to weight.
