# Supplementary material for: Cross-sectional study of influenza trends and costs in Malaysia between 2016 and 2018
Source: PLoS One. 2024 Mar 22;19(3):e0301068. doi: 10.1371/journal.pone.0301068 (PMC10959333; doi:10.1371/journal.pone.0301068)
Supplement: S3 Table — (PDF) [file pone.0301068.s006.pdf]

S3 Table. Costs of Severe Acute Respiratory Infection Treatment at Four Malaysian Treatment Sites.

DRG codes	PPC (MYR)	Number of cases (*n* = 75,225)			Total price (MYR)		
		**HCTM UKM**	**MOH**	**UMMC and KPJ**	**HCTM UKM**	**MOH**	**UMMC and KPJ**
**Respiratory infection**							
4581	4,088.30	2,683	61,774	1,888	10,968,908.90	252,550,644.20	7,718,710.40
4582	3,876.39	178	4,164	99	689,997.42	16,141,287.96	383,762.61
4583	5,400.95	121	3,470	60	653,514.95	18,741,296.50	324,057.00
**Total**		2,982	69,408	2,047	12,312,421.27	287,433,228.66	8,426,530.01
**ENT and orbital infection**							
3501	2,356.40	NA	NA	511	NA	NA	1,204,120.40
3502	2,549.70	NA	NA	65	NA	NA	165,730.50
3503	3,325.10	NA	NA	31	NA	NA	103,078.10
**Total**				607			1,472,929.00
**Blood disorder**							
16501	2,505.86	NA	NA	93	NA	NA	233,044.98
16502	3,211.70	NA	NA	57	NA	NA	183,066.90
16503	4,664.50	NA	NA	61	NA	NA	284,534.50
**Total**				211			700,646.38

ENT, ear, nose, and throat; DRG, diagnosis-related group; HCTM UKM, Hospital Canselor Tuanku Muhriz UKM; KPJ, KPJ Hospital; MYR, Malaysian ringgit; MOH, Ministry of Health; NA, not applicable; PPC, price per case; SARI, severe acute respiratory infection; UMMC, University Malaya Medical Center.
